# Manipulation of targeted protein degradation in plant biology

**DOI:** 10.1042/BST20230939

**Published:** 2025-04-09

**Authors:** Marcela Rojas-Pierce, Sebastian Y. Bednarek

**Affiliations:** 1 Department of Plant and Microbial Biology, North Carolina State University, Raleigh, NC, U.S.A.; 2 Department of Biochemistry, University of Wisconsin-Madison, Madison, WI, U.S.A.

**Keywords:** degron, plant biology, proteolysis, targeted protein degradation, ubiquitination, ubiquitin ligases

## Abstract

Inducible protein degradation systems are an important but untapped resource for the study of protein function in plant cells. Unlike mutagenesis or transcriptional control, regulated degradation of proteins of interest allows the study of the biological mechanisms of highly dynamic cellular processes involving essential proteins. While systems for targeted protein degradation are available for research and therapeutics in animals, there are currently limited options in plant biology. Targeted protein degradation systems rely on target ubiquitination by E3 ubiquitin ligases. Systems that are available or being developed in plants can be distinguished primarily by the type of E3 ubiquitin ligase involved, including those that utilize Cullin-RING ligases, bacterial novel E3 ligases, and N-end rule pathway E3 ligases, or they can be controlled by proteolysis targeting chimeras. Target protein ubiquitination leads to degradation by the proteasome or targeting to the vacuole, with both pathways being ubiquitous and important for the endogenous control of protein abundance in plants. Targeted proteolysis approaches for plants will likely be an important tool for basic research and to yield novel traits for crop biotechnology.

## Introduction

Control of protein abundance is essential for understanding complex biological pathways. Over the past decade, significant methodologies have been developed for the inducible degradation or stabilization of target proteins of interest (POIs) in animal cells and yeast [1,2]. These approaches primarily leverage the cellular proteolytic pathways inherent in eukaryotic organisms, enabling researchers to manipulate protein abundance at the post-translational level. The two principal proteolytic systems in eukaryotic cells, the ubiquitin-proteasome system (UPS) and the vacuolar degradation pathway, serve as the backbone for many of these innovative techniques [3,4]. The UPS controls protein abundance as part of eukaryotic developmental and physiological pathways and is particularly important and ubiquitous in a variety of plant signaling pathways including hormone and stress responses [5–8]. Controlled target protein ubiquitination represents an untapped strategy for manipulation of biological pathways in plants, with potential applications to basic functional studies and agriculture biotechnology [9]. However, relative to gene expression control systems, this approach, to date, has not been exploited in plants because of the limited availability of tools to control the ubiquitination of protein targets. We describe recent approaches that begin to show the feasibility of using regulated protein degradation for manipulation of plant traits and discuss remaining challenges and opportunities.

### Mechanisms for protein ubiquitination and degradation

Ubiquitination involves the covalent attachment of ubiquitin molecules to a substrate, which signals proteins for degradation. Protein ubiquitination is an ATP-dependent enzymatic process that proceeds through a sequential cascade involving an E1 ubiquitin-activating enzyme, an E2 ubiquitin-conjugating enzyme, and an E3 ubiquitin ligase. Depending on the length and linkage type of the ubiquitin chain, ubiquitinated proteins may be targeted for degradation by the 26S proteasome or the vacuole [10–12]. E3 ubiquitin ligases are primarily responsible for the specificity of substrate recognition and the transfer of ubiquitin onto their target proteins [13,14], and are typically categorized into three main types: Really Interesting New Gene (RING), Homologous to the E6AP Carboxyl Terminus (HECT), and RING-IBR-RING (RBR) E3 ligases [11]. Cullin-RING ligases (CRLs) are multisubunit RING E3 ligases formed by a Cullin scaffold protein, a RING-box protein (RBX) that binds to the E2, and a substrate receptor. CRL substrate binding can be mediated by one or more subunits. A subcategory of CRL complexes is the Skp1-Cullin1-F-box complex that contains the Skp1 adapter protein and various F-box proteins that serve as the substrate recognition component. For example, in the case of the auxin receptor, substrate binding involves the F-box protein TIR1 and the Skp1 adaptor that binds to TIR1 and a Cullin scaffold [15]. In addition, N-recognins, or UBR Box E3 ligases, are another class of E3 ligases that ubiquitinate proteins and target them for degradation via the N-end protein pathway [10,16–18]. Both HECT- and RING-type N-recognins have been identified [19]. There are more than 1,400 E3 ligases in Arabidopsis [8] compared with ~600 in humans [20], which underscores the complexity and potential of ubiquitination pathways in plants.

The specificity of the UPS is largely determined by the presence of degrons—minimal sequences or structural motifs that promote interaction between a protein target and an E3 ligase. N-degrons are characterized by specific destabilizing amino acid residues at the N-termini of proteins, which direct their recognition by E3 ubiquitin ligases known as recognins [10]. Similarly, C-degrons, located at the C-terminus, are recognized by CRL-type E3 ligases [10]. In addition, degrons may be located within the internal regions of proteins [18]. A particularly important example of internally located degrons in plant proteins is the ligand-induced degrons (LIDs), which promote plant hormone-dependent association of CRL E3 ligases and their targets. These include LIDs involved in auxin and jasmonate signaling [10]. Elucidation of the mode of action of the various classes of E3 ligases and their associated degrons has led to the development of new strategies for targeted protein manipulation and therapeutic intervention in non-plant systems.

Below, we review various systems for targeted protein degradation currently available in plants, as well as pioneering approaches that, although not suitable for plant research, provide valuable design principles for future applications. Key factors to consider in the design and/or application of degron systems include (1) the type of E3 ubiquitin ligase involved, (2) a mechanism for target recognition by the E3 ligase, which in most cases involves specific protein–protein interaction domains or degrons, and (3) a mechanism for induction of E3 ligase activity or E3 target interaction (Table 1). We distinguish current technologies by the type of E3 ligase involved including those that utilize CRL-based E3 ubiquitin ligases, bacterial novel E3 ligases (NELs), and those that take advantage of the N-end rule pathway. In addition, we discuss the potential of using proteolysis targeting chimeras (PROTACs) which, in principle, could be designed to work with either type of E3 or E2, as well as the potential engineering of E3 ligases for targeted protein degradation via the vacuolar system. E3 target interaction may be initiated by intrinsic protein–protein interaction domains, binding to molecular glues that enhance their binding affinity for substrates (e.g. auxin binding to TIR1) or binding of PROTACs that bring an E3 and a target closer together [2].

**Table 1:** Comparison of targeted protein degradation systems in various organisms with emphasis on plants.

Degron system	Type of E3 ligase	Degron	Target recruitment	Inducer	Tested in plants?
AID/AID2	Chimeric CRL E3 ligase (TIR1)	Aux/IAA17 (Aux/IAA17 DII domain)	TIR1 F-box protein	Auxin/NAA	No
deGradFP	Chimeric CRL E3 ligase (NSlmb-VhhGFP4)	GFP	Anti-GFP nanobody Vhh4	Expression of NSlmb-VhhGFP4	Arabidopsis, Nicotiana
FKBP-FRB based	Chimeric CRL E3 ligase (FKBP-Culin)	FRB	FKBP-FRB binding	Rapamycin	Red algae
E3 DART	Novel E3 ligase (SspH1)	HR1b peptide	HR1b-E3 DART (LRR) binding	DEX-inducible expression of E3 DART	Nicotiana
ts-degron	N-end E3 ligases (likely UBR1 in S. cerevisiae but not tested)	R-DHFR N-degron	N-degron recognition	37°C	No
lt-degron	N-recognin PRT1 or PRT6 in Arabidopsis	R/F-DHFR^T39A/E173D^ N-degron	N-degron recognition	29°C	Arabidopsis, Nicotiana
FKBP-derived DD	Unknown	FKBP^F36VL106P^	Unknown	Shield (stabilization)	Arabidopsis
RDDK	N-end E3 ligases (likely but not tested)	RDDK (R-FKBP^F36VL106P^-K)	N-degron recognition (likely but not tested)	Shield (stabilization)	Arabidopsis, wheat, rice
ecDHFR	Unknown	ecDHFR	Unknown	Trimethoprim (stabilization)	No
TIPI	N-end E3 ligases (likely but not tested)	R-N-degron	N-degron recognition (likely but not tested)	Expression of TEV protease	Arabidopsis
PROTAC	Endogenous E3 ligase	PROTAC binding site	PROTAC binding	PROTAC	No

CRLs, Cullin-RING ligases. DEX, dexamethasone. ecDHFR, dihydrofolate reductase from *E. coli*. E3 DART, E3-targeted degradation of plant protein. FKBP, FK506-binding protein. FRB, FKBP-rapamycin-binding. NAA, naphthaleneacetic acid. PROTAC, proteolysis targeting chimera. TEV, tobacco etch virus. TIPI, TEV protease-induced protein instability.

### Targeted protein degradation with CRL E3 ligases

**Figure 1:**
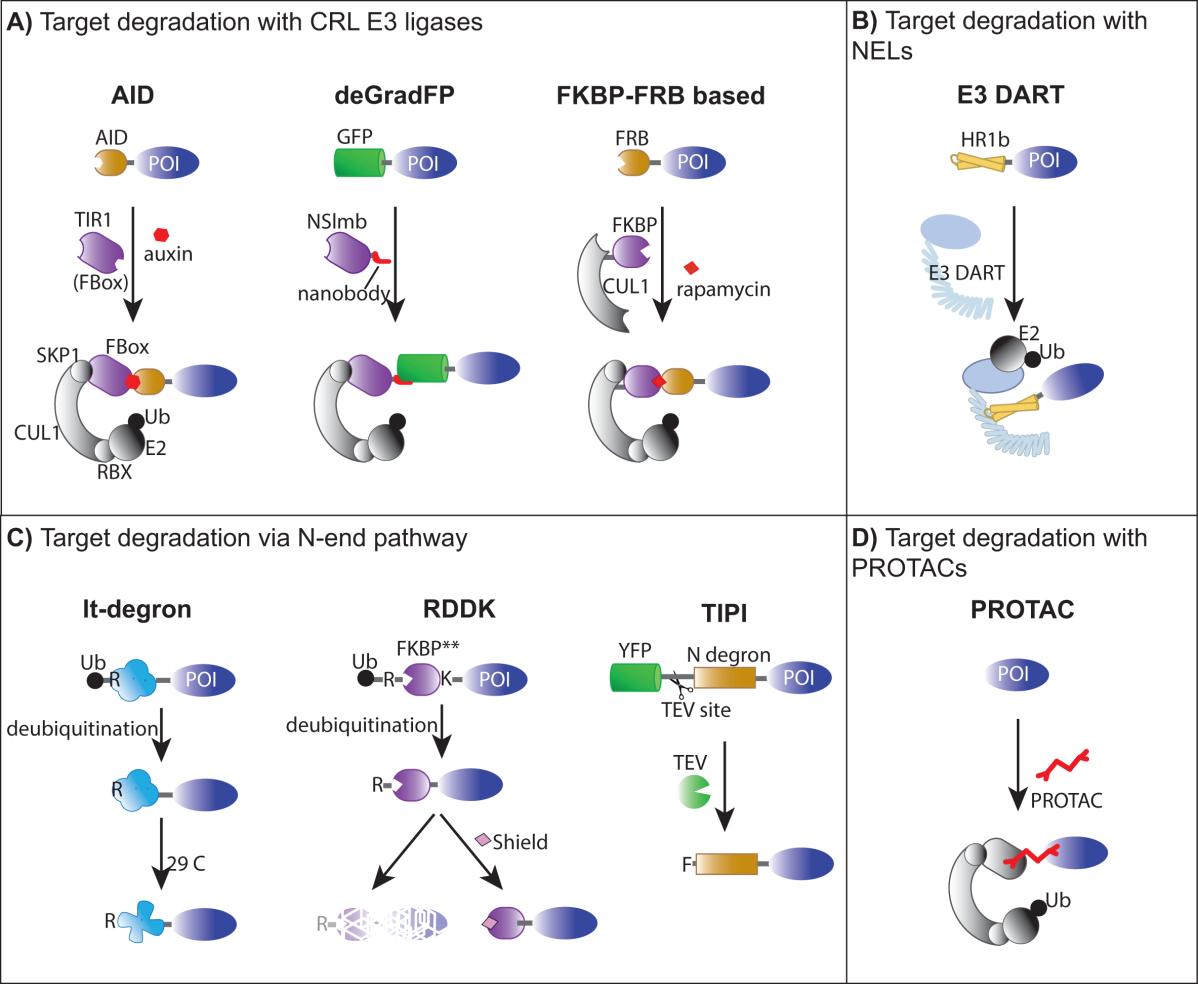
Strategies for targeted protein degradation in various organisms including plants. For each system, a protein of interest (POI) is fused to a protein domain that controls the mode of recruitment to a degradation pathway. Endogenous components of E3 ligase complexes and E2 ubiquitin-conjugating enzymes needed for formation of chimeric complexes are shown in gray. Protein components that need to be co-expressed via transformation are shown in color next to the arrows. Ligands that promote protein–protein interactions or regulate protein stability are indicated. Resulting ubiquitination and degradation of POIs at the end of each pathway has been omitted for clarity. For details on each domain, see the text. Ub: Ubiquitin. **(A)** Degron systems that use CRL E3 ligases for protein degradation include the auxin-induced degron (AID, not used in plants), deGradFP, and a rapamycin-inducible protein knockdown. Assumed subunits of CRL E3 ligases that assemble with heterologous F-box proteins include Skp1, CUL1, and RBX. **(B)** Degradation systems that use microbial novel E3 ligases (NELs). The E3 DART system targets POI degradation by tagging it with the HR1b domain and co-expression of the E3 DART ligase. E3 DART is expected to associate with endogenous E2 ligases to catalyze ubiquitination. **(C)** Degradation systems that initiate ubiquitination via the N-end rule pathway. These systems use endogenous deubiquitylases or a protease to expose an unstable residue, R or F, at the N-terminus of the POI triggering ubiquitination by the N-end rule pathway. In the lt-degron, the R residue is protected by protein folding at low temperatures. For the RDDK system, the deubiquitinated protein is ubiquitinated and degraded, but the application of the Shield ligand prevents ubiquitination and promotes protein accumulation. KBPB^**^ corresponds to the FKBP12^F36V L106P^ variant. In the TIPI system, the unstable F residue is exposed after cleavage by a TEV protease. **(D)** PROTACs. A specific bifunctional ligand that binds to the POI and an endogenous E3 ligase is used to promote POI degradation. CRL, Cullin-RING ligases; E3 DART, E3-targeted degradation of plant protein; PROTACs, proteolysis targeting chimeras; TEV, tobacco etch virus.

Several protein degrons utilize CRL-type E3 ligases for ubiquitination of protein targets (Figure 1A). Given the modular nature of CRL complexes, these CRL-based degradation systems typically involve the expression of a small E3 ligase subunit, such as an F-box protein, that provides specificity for target recruitment. These systems rely on the assembly of chimeric E3 ligases including the heterologous expression of an F-box protein (in most cases), which assembles with the endogenous complement of CRL ligase subunits (CULLIN1, Skp1, and Rbx). The highlight of these systems includes the auxin-inducible degron (AID and AID2) systems [21,22] and the coronatine-induced degron (pJAZ) based on the jasmonic acid receptor COI1 [23]. The original AID design was based on the activity of the TIR1 auxin receptor [21]. TIR1 is an F-box protein that associates with CULLIN1 and SKP1 to form the SCF^TIR^ E3 ubiquitin ligase complex [24]. Neither auxins nor TIR1 are present outside of plants such that the expression of TIR1 and a fusion of a protein target with the Arabidopsis Aux/IAA protein IAA17 (AID degron) result in auxin-induced ubiquitination of AID target fusions in yeast [21]. This indicates that TIR1 assembles into a functional chimeric CRL E3 complex together with endogenous yeast Spk1 and Cul1 homologues. In this system, 30–60 minutes of incubation with the synthetic auxin 1-naphthaleneacetic acid (NAA) was sufficient for AID target fusion degradation [21]. Shorter AID degrons, alternative F-box auxin receptors, and bump–hole variations of TIR1 and auxin are just a few variations of the AID system that have decreased basal degradation and increased sensitivity [22,25,26]. Auxin-based degron systems have been applied to many organisms including human cells and mice [reviewed in 1], and similar approaches have been developed using the jasmonic acid receptor COI1 and coronatine [23]. AID and pJAZ are inducible by the cognate hormone such that targeted protein degradation is solely controlled by post-translational mechanisms. The adoption of an auxin or coronatine degron system in plants is likely to be unsuccessful as auxin and coronatine treatments result in major changes at the transcriptional and post-transcriptional levels. Even the potential use of the orthogonal hormone–receptor pair, convex IAA (cvxIAA), and concave TIR1 (ccvTIR1), which was recently developed by a bump and hole approach [27], could be problematic as ccvTIR1 is likely to interact with most TIR1 targets. Nonetheless, the AID and pJAZ systems highlight the feasibility of high-jacking endogenous ubiquitin ligases by providing heterologous F-box subunits.

The degrade Green Fluorescent Protein (deGradFP) system also relies on ubiquitination via chimeric CRL E3 ligases and has been used to target GFP-bearing fusions in *Nicotiana benthamiana* [28] and Arabidopsis [29,30]. The deGradFP system uses a fusion of the N-terminus of the *Drosophila* F-box protein Slmb to the anti-GFP nanobody VhhGFP4. The Slmb fragment contains an F-box domain for incorporation into CRL E3 ligase complexes in model metazoan systems. The anti-GFP nanobody functions as a protein–protein interaction domain to recruit GFP-bearing proteins to the chimeric CRL E3 ligase [31–33]. The expression of the F-box protein under control of tissue-specific and inducible promoters allows for an efficient depletion of target proteins in a temporal and spatial manner [34–36]. Following its validation in Nicotiana transient assays [28], deGradFP was expressed under an egg cell-specific promoter to target ERECTA-YPet fusions in Arabidopsis zygotes [30]. The same system was also used to induce degradation of WUSHEL (WUS) in the Arabidopsis shoot apical meristem by the expression of the deGradeFP construct under the ethanol-inducible pAlc promoter [29]. WUS-linker-GFP levels were reduced after 24 h of induction as detected by fluorescence microscopy. These examples underscore the power of cell inducible or tissue-specific expression of E3 ligases for the studies of POIs with discrete ranges of expression. Compared with the rapid ligand-induced auxin or coronatine-induced degradation systems (Table 1), the use of deGradFP is dependent on transcriptional regulation of the F-box protein and, thus, is not ideal for highly dynamic processes.

Another CRL-based protein degradation system was recently developed in the red algae *Cyanidioschyzon merolae* [37]. This system exploits the activity of rapamycin, a small ligand that promotes the binding of the mammalian target of rapamycin (mTOR) kinase to FK506- and rapamycin-binding protein (FKBP). The target fusion protein contains the human FKBP-rapamycin-binding (FRB) domain of mTOR and associates with FKBP-tagged human Cullin1 (CUL1) in cells treated with rapamycin. In algae co-expressing Venus-FRB and FKBP-CUL1, target protein degradation was detected 2 h after rapamycin application. One challenge of this system is that the FRB domain confers varying levels of instability to protein targets [38], even in the absence of the FKBP-CUL1 fusion [37]. As a result, establishing a reliable ‘stable’ untreated baseline may be difficult. Moreover, transcriptome analyses raise concerns regarding the specificity of the chimeric E3 ligase as it appears to recognize protein targets beyond the POI-FRB protein. Furthermore, while this system is likely to contribute to protein function studies in red algae and other organisms where RNA silencing strategies are unavailable [37], it is likely unsuitable to rapamycin-sensitive plants including Arabidopsis [37,39,40].

### Targeted protein degradation with NEL

The recently developed E3-targeted degradation of plant proteins (E3 DART) system [41] (Figure 1B) uses an NEL from *Salmonella* that highjacks the human ubiquitination system during infection. *Salmonella*-secreted protein H1 (SspH1) is a protein effector that targets human protein kinase N1 (PKN1) for ubiquitination [42]. Unlike degradation systems that use F-box proteins and require assembly of multimeric CRL E3 ligases (e.g. AID, deGradFP, Figure 1A), E3 DART is a single polypeptide containing both the leucine-rich repeat (LRR) target recruitment and E3 catalytic (NEL) domains of SspH1. The LRR domain binds specifically to the HR1b domain of PKN1, and therefore, HR1b is used as a degron to target POIs for ubiquitination with E3 DART [41]. Transient expression in Nicotiana demonstrated that E3 DART targets HR1b-protein fusions for degradation via the proteosome. Since both SspH1 and its recognition target, the HR1b domain from PKN1, are absent from plants, there is limited risk of targeting endogenous plant proteins for degradation [41]. A synthetic dexamethasone (DEX)-inducible transactivation system [43] was used to control the expression of E3 DART. Induction of E3 DART was detected 3 h after DEX treatment in agroinfiltrated Nicotiana leaves, and this increase was concomitant with the degradation of HR1b-tagged fusions. However, the DEX induction system was not effective in Arabidopsis [41], mostly due to weak induction of E3 DART ligase expression, which is consistent with limitations of plant inducible gene expression systems including silencing [44–46]. Nonetheless, the robustness of the E3 DART system could be taken advantage in specific designs by the expression of the E3 DART ligase under cell-type specific promoters, other induction systems or in other plant species where DEX induction is more effective. A similar strategy, named ubiquibodies, was developed for mammalian cells but only used the NEL domain of SspH1 and other microbial E3 ligases. GFP target binding of ubiquibodies was controlled with the fibronectin type III (FN3) monobody against GFP [47]. Ubiquibodies have not been tested in plants, but deletion of the LRR domain of SspH1 resulted in unstable E3 DART protein in Nicotiana (Huang and Rojas-Pierce, unpublished). One major drawback of the current E3 DART system is the fact that it relies on the induction of E3 ligase expression rather than activation at the post-translational level.

### Targeted protein degradation via the N-end rule pathway

Several protein degradation systems take advantage of the N-end rule pathway (Figure 1C). In all cases described below, protein ubiquitination is induced by the formation of a destabilizing N-degron, but very few specific E3 ligases involved in their recognition have been identified. Early versions of these systems include those activated by high temperature. The first temperature-sensitive degron (ts-degron) was developed from a ubiquitin-Arginine-fused variant of the mouse dihydrofolate reductase (Ub-R-DHFR), which is stable in yeast at 23°C and rapidly degraded when cells were incubated at the restrictive temperature of 37°C [48]. In this system, the ubiquitin moiety would be quickly cleaved by endogenous deubiquitylases resulting in exposure of an N-terminal R residue, which would be subject to protein degradation via the N-end rule pathway [17]. In the case of Ub-R-DHFR-tagged proteins, this new N-degron was destabilizing only at the restrictive temperature [48]. It was proposed that protein folding at ambient temperatures made the destabilizing N-end R-degron inaccessible for N-end rule pathway recognition enzymes, while the high temperature resulted in protein unfolding, N-degron recognition, and subsequent ubiquitination. The 37°C requirement makes this system impractical to plants. A low-temperature degron (lt-degron) was later developed and included two different mutations of DHFR (T39A/E173D), which shifted the permissible temperature to 16°C and the restrictive temperate to 29°C [49]. Depending on whether the residue at the N-terminus was a Phe or an Arg, lt-degron recognition in Arabidopsis was dependent on the plant N-recognins PROTEOLYSIS1 (PRT1) or PRT6, respectively [49]. The system was tested in Nicotiana and Arabidopsis with multiple target proteins including CONSTANS (CO), TRANSPARENT TESTA GLABRA1 (TTG1), GUS, and GFP with degradation being observed after 2–3 h of incubation at the restrictive temperature [49]. A challenge of the lt-degron is the required 29°C incubation for full degradation of POIs [49], which can have significant effects on the pathway of study. Nevertheless, this system has been successfully used to induce cellular toxicity in a temperature-controlled manner in Arabidopsis by fusing the lt-degron to a bacterial RNAse barnase [50]. Thus, biotechnology applications compatible with protein degradation at high temperature could potentially be developed using this system.

Systems that use small ligands to control protein abundance via the N-end rule pathway were developed using protein destabilization domains (DDs). Protein fusions to DDs are constitutively degraded in the absence of cognate ligands but are stabilized after binding to them. The first DD was developed for fibroblasts based on variants of human FK506- and rapamycin-binding protein FKBP12 (FKBP12^F36V^) [51] that, in contrast to wildtype FKBP12, bind specifically to rapamycin derivatives that are ineffective in mTOR binding. A second mutation in FKBP12, L106P, was identified by random mutagenesis and resulted in tight control of protein stability by the rapamycin derivative Shield-1 [51]. Fusions of protein targets with the FKBP12^F36V L106P^ tag were stable in the presence of Shield-1 but quickly degraded when the ligand was removed [51]. This system was effective in mammalian cells and other species including *Plasmodium falciparum* [52] and *Toxoplasma gondii* [53], but it was found to only partially destabilize a POI in the absence of Shield in Arabidopsis plants [54]. Further modifications of FKBP12^F36V L106P^ resulted in enhanced degradation of tagged POIs in plants in the absence of ligand [54]. This included a similar strategy as described above for the ts-degron [48] in which Ub-R was appended to the N-terminus, with the addition of a Lysine (K) at the C-terminus of FKBP12^F36V L106P^ DD (RDDK). Cleavage of the N-terminal Ub exposes the N-terminal R residue triggering efficient degradation of RDDK-tagged POI by the N-end rule pathway [55] in the absence of Shield-1 in Arabidopsis [54], rice, and wheat [56]. The effective window of RDDK-tagged protein accumulation after Shield-1 treatment was 1 h in Arabidopsis but up 6 h in rice [56]. Remarkably, Shield-1 was effective even when sprayed on leaves of intact rice and wheat plants with significant results [56].

A similar DD strategy to control protein abundance was based on a modified dihydrofolate reductase from *Escherichia coli* (ecDHFR) and the small ligand trimethoprim (TMP). Like the FKBP-Shield-1 system, ecDHFR DD is stable only in the presence of TMP. This system was tested in fibroblasts and rat brains [57], and a modified version was developed for *Caenorhabditis elegans* which grows at lower temperatures (15–25°C) [58]. RDDK has shown great promise for control of protein stability in plants, but the drawbacks include the cost of Shield-1, being functional only as a N-terminal fusion [51,56]. DHFR-based DD systems are effective for both N- and C-terminal fusions, TMP is less expensive, and DHFR mutants that are effective at the temperatures appropriate for plant growth are available [57,58], but no reports for its application in plants are available to date, and possible secondary effects of TMP have not been tested. Another potential issue is that the RDDK- and DHFR-based DD approaches are likely not suitable for the study of essential proteins in plants as they promote the degradation of their fusion POIs in the absence of their respective stabilizing ligands.

The tobacco etch virus (TEV) protease-induced protein instability (TIPI) degron system harnesses protease cleavage to generate an unstable N-degron to induce degradation of a protein target via the N-end rule pathway [59]. In this system, a POI is flanked at the N-terminus by a 7 aa cleavage site for the TEV protease and an N-terminal degron domain. TEV cleavage generates a phenylalanine (F) destabilizing residue flanking the N-terminal degron motif on the tagged POI, resulting in its recognition by the N-end rule pathway and subsequent ubiquitination [59]. This system was used in Arabidopsis meristems to control the abundance of an N-terminal yellow fluorescent protein (YFP)-tagged WUS fusion protein in which the linker between YFP and WUS contained the 7 aa TEV cleavage site followed by an N-terminal degron domain (YFP-WUS^TEVrs^) [60]. The full-length YFP-WUS^TEVrs^ protein localizes to the nucleus, but co-expression with the TEV protease resulted in protein cleavage as visualized by the accumulation of the cytosolic free YFP marker. The expression of the TEV protease under a stem-cell specific promoter was used to degrade WUS in a cell-specific manner. The efficiency of TEV cleavage and WUS degradation was not quantified, however, but the authors indicate that in some plants, the TEV protease was not able to fully process the WUS fusion proteins as reported by the accumulation of cytosolic YFP. Further characterization of the efficiency of the TEV protease and the N-degron of TIPI is needed for the application of TIPI to a variety of plant tissues.

### Targeted degradation with PROTACs

Molecular glues and PROTAC protein degraders are small synthetic molecules or peptides that promote the interaction between a POI and an endogenous E3 ligase to promote the ubiquitination of the target [20]. Molecular glues are small ligands that bind to either the E3 ligase or the target, but not both, and alter the binding affinity of E3-target complex, an example being auxin which promotes the interaction between TIR1 and the degron motif of AUX/IAA proteins [2]. PROTACs are cell-permeable, small heterobifunctional chemical probes consisting of two ligands joined by a linker. One of the PROTAC ligands (also called warheads) binds selectively with the target POI, while the other binds an E3 ligase (Figure 1D). PROTACs, therefore, bring the POI into close proximity with the E3 ligase complex promoting its ubiquitination and subsequent degradation [61]. The development of PROTACs was catalyzed by discovering that Cereblon (CRBN), the target receptor of an E3 CRL ligase, binds to thalidomide, a drug used against multiple myeloma. Binding of thalidomide results in ubiquitination of two transcription factors with proliferative activity [2,62]. Thalidomide was later conjugated via a linker to a binding moiety for a POI target to generate the first PROTAC molecule [2,63]. Several PROTACs are currently in clinical trials including two that use human Cereblon (CRBN) as the E3 ligase [20] and have demonstrated feasibility for human therapies [20]. The advantage of PROTACs is that they harness the function of endogenous E3 ligases and can be provided exogenously. The development of PROTACs is highly dependent on the identification of robust chemical probes that bind specifically to only one (or a few) highly expressed E3 ligases so that ubiquitination of a POI can be achieved in diverse cell types and developmental stages. Thus, the identification of PROTACs that bind to specific POIs is also potentially a bottleneck to this promising technology and a particular challenge in plants at this stage given the limited information regarding the structure of many plant POIs. With the rapid development of protein structure prediction and ligand docking tools from AlphaFold [64,65] and other IA technologies, new leads of PROTAC warheads toward plant targets may be on the horizon. Commercial development of PROTACs is in the horizon [66], but future research will determine how effective they will be for plant research and biotechnology. Being the only targeted proteolysis system that does not depend on transgenic expression of target protein fusions and/or catalytic enzymes, PROTACs are highly attractive as a tool for regulated degradation of plant target proteins in a wide number of plant species. Nonetheless, the expression of heterologous E3 ligases such as CRBN and control by a well-characterized PROTAC may be a possibility for future protein degraders in plant research.

## Future prospects

Many challenges still exist for inducible protein degradation systems in plants, and more research is needed for their optimization. E3 DART, PROTACs, and DDRK represent the first steps toward functional applications of protein degraders. The development of multiple synthetic approaches will be necessary to provide flexibility to multiple plant species and tissues with different requirements for ligand uptake, plant transformation, and ease of validation. Recent identification of novel plant degrons [10] may yield further development of new strategies to control protein destabilization in plants. Protein degradation systems described here involve an E3 ligase. However, it may also be feasible to harness E2 conjugating enzymes for targeted protein degradation. Previously, it was demonstrated that plant E2s, when fused to target-binding domains, were able to functionally ubiquitinate protein targets *in vitro* [67]. A recent approach has shown success with the use of E2-target-binding fusions in human cells [68], underscoring the potential of yet another approach for targeted ubiquitination and degradation of POIs in plants. All examples of targeted protein degradation in plants so far involve cytoplasmic or nuclear proteins that are accessible to the UPS. In the case of membrane proteins, it may be possible to use similar approaches that rely on targeting of proteins to the vacuole via endocytic pathways [69]. Moreover, whole organelles could potentially be targeted for degradation in the vacuole via autophagy as recently demonstrated in animal cells [70]. Enhanced validation of existing approaches, coupled with the development of innovative tools, is likely to facilitate their effective application in studying plant POIs in the laboratory and advancing agronomic practices in the field.

**Perspectives**

- Targeted protein degradation is an important toolbox for functional studies in eukaryotes, but facile systems have been out of reach for plant biologists.
- New tools have recently been developed to target proteins of interest for degradation via the proteosome in plants. Successful and robust application of these strategies remains to be developed for multiple plant species including crops and for a variety of organs and tissues.
- Future development of current strategies or new strategies for induced target protein ubiquitination may contribute to functional studies of essential genes or to the design of new traits for biotechnology applications.

## Abbreviations

CRLs: Cullin-RING ligases
DEX: dexamethasone
E3 DART: E3-targeted degradation of plant protein
FKBP: FK506-binding protein
FRB: FKBP-rapamycin-binding
NAA: naphthaleneacetic acid
PROTAC: proteolysis targeting chimera
TEV: tobacco etch virus
TIPI: TEV protease-induced protein instability
ecDHFR: dihydrofolate reductase from *E. coli*
